# Identification and validation of novel biomarkers associated with immune infiltration for the diagnosis of osteosarcoma based on machine learning

**DOI:** 10.3389/fgene.2023.1136783

**Published:** 2023-09-04

**Authors:** Yuqiao Ji, Zhengjun Lin, Guoqing Li, Xinyu Tian, Yanlin Wu, Jia Wan, Tang Liu, Min Xu

**Affiliations:** ^1^ Department of Orthopedics, The Second Xiangya Hospital, Central South University, Changsha, Hunan, China; ^2^ Department of Critical Medicine, The Second Xiangya Hospital, Central South University, Changsha, Hunan, China

**Keywords:** osteosarcoma, machine learning, immune cell infiltration, diagnosis, hub genes

## Abstract

**Objectives:** Osteosarcoma is the most common primary malignant tumor in children and adolescents, and the 5-year survival of osteosarcoma patients gained no substantial improvement over the past decades. Effective biomarkers in diagnosing osteosarcoma are warranted to be developed. This study aims to explore novel biomarkers correlated with immune cell infiltration in the development and diagnosis of osteosarcoma.

**Methods:** Three datasets (GSE19276, GSE36001, GSE126209) comprising osteosarcoma samples were extracted from Gene Expression Omnibus (GEO) database and merged to obtain the gene expression. Then, differentially expressed genes (DEGs) were identified by limma and potential biological functions and downstream pathways enrichment analysis of DEGs was performed. The machine learning algorithms LASSO regression model and SVM-RFE (support vector machine-recursive feature elimination) analysis were employed to identify candidate hub genes for diagnosing patients with osteosarcoma. Receiver operating characteristic (ROC) curves were developed to evaluate the discriminatory abilities of these candidates in both training and test sets. Furthermore, the characteristics of immune cell infiltration in osteosarcoma, and the correlations between these potential genes and immune cell abundance were illustrated using CIBERSORT. qRT-PCR and western blots were conducted to validate the expression of diagnostic candidates.

**Results:** GEO datasets were divided into the training (merged GSE19276, GSE36001) and test (GSE126209) groups. A total of 71 DEGs were screened out in the training set, including 10 upregulated genes and 61 downregulated genes. These DEGs were primarily enriched in immune-related biological functions and signaling pathways. After machine learning by SVM-RFE and LASSO regression model, four biomarkers were chosen for the diagnostic nomogram for osteosarcoma, including ASNS, CD70, SRGN, and TRIB3. These diagnostic biomarkers all possessed high diagnostic values (AUC ranging from 0.900 to 0.955). Furthermore, these genes were significantly correlated with the infiltration of several immune cells, such as monocytes, macrophages M0, and neutrophils.

**Conclusion:** Four immune-related candidate hub genes (ASNS, CD70, SRGN, TRIB3) with high diagnostic value were confirmed for osteosarcoma patients. These diagnostic genes were significantly connected with the immune cell abundance, suggesting their critical roles in the osteosarcoma tumor immune microenvironment. Our study provides highlights on novel diagnostic candidate genes with high accuracy for diagnosing osteosarcoma patients.

## 1 Introduction

Osteosarcoma is one of the most common bone malignancies that mainly affect children and adolescents (Ritter and Bielack, 2010; Gill and Gorlick, 2021). Despite advances in the development of treatment approaches, improving the clinical outcomes of patients with osteosarcoma is still largely challenging (Chen Y. et al., 2021; Meltzer and Helman, 2021). Notably, the genomic complexity and instability of osteosarcoma have become the major hinder to successful treatment (Kansara et al., 2014; Gianferante et al., 2017). It is necessary to optimize the early detection, treatment progress, and prognosis prediction of osteosarcoma based on molecular genetics. Hence, developing novel and reliable biomarkers to complement and improve current osteosarcoma screening strategies is urgently warranted. Notably, the tumor immune microenvironment has gained increasing attention, and complex tumor immune microenvironment components can contribute to the tumor heterogeneity and multifaceted mechanisms of osteosarcoma progression and metastasis (Zhou et al., 2020; Yu et al., 2021; Marchais et al., 2022). For instance, a previous single-cell RNA study has reported that the infiltration of proinflammatory FABP4 macrophages is detected in lung metastatic osteosarcoma lesions, and TIGIT (T cell immune receptor with immunoglobulin and ITIIM domain) blockade treatment significantly facilitates the cytotoxicity effects of the primary CD3^+^ T cells with a high proportion of TIGIT + cells against osteosarcoma (Zhou et al., 2020). Hence, identifying immune-related gene signatures can facilitate the diagnosis of patients with osteosarcoma and the elucidation of the general mechanisms of osteosarcoma initiation and progression.

Nowadays, comprehensive bioinformatics analysis and microarray technology have been widely applied to explore novel specific disease-related genes and their biological functions, thus helping the early diagnosis of diseases and illustrating the underlying mechanisms of disease occurrence and development (Perez-Iratxeta et al., 2007; Werner, 2008; Gong et al., 2018). Machine learning is an emerging field of artificial intelligence, which can identify future trends and predict the results of existing data (Deo, 2015; Greener et al., 2022). Machine learning has broad applications in biological medicine, and can effectively explore prospective biomarkers and therapeutic targets, and potential mechanisms for various human diseases (Bigorra et al., 2019; Chen Z. et al., 2021; Liu et al., 2021). For instance, five immune-associated diagnostic genes (*ITGAL*, *CXCL16*, *MORF4L2*, *SPRY2*, and *BEX2*) have been identified to diagnose aortic valve calcification patients with metabolic syndrome by employing bioinformatics analysis and machine learning algorithms (Zhou et al., 2022). Recently, several studies have explored novel diagnostic and prognostic biomarkers in osteosarcoma by comprehensive bioinformatics analysis. For instance, Li et al. (2022) have constructed a model based on six machine learning (ML) algorithms for the prediction of lymph node metastasis, and T and M stage, surgery, and chemotherapy have been regarded as independent risk factors. A recent study has constructed a prognosis model based on ferroptosis-related genes for osteosarcoma patients by univariate COX regression and LASSO regression (Huang et al., 2023). However, there has limited research focusing on the identification and validation of diagnostic biomarkers associated with immune signatures for patients with osteosarcoma by combining machine learning and bioinformatics methods, as well as further illustrating the prognostic value of these diagnostic candidates.

In the present study, we obtained three osteosarcoma datasets from the GEO database, which were further merged and divided into two sets. After identifying DEGs in the training set, machine learning algorithms were employed to further select key diagnostic candidates. We also investigated the immune landscape in osteosarcoma and explored the relationship between these potential diagnostic biomarkers and immune cell infiltration. Besides, we also validated the expression pattern of these biomarkers *in vitro*.

## 2 Materials and methods

### 2.1 Data acquisition and process

Three raw datasets (GSE19276, GSE36001, GSE126209) comprising osteosarcoma samples were extracted from the GEO database (https://www.ncbi.nlm.nih.gov/geo/). The GSE19276 (comprising 44 osteosarcoma samples and 5 normal samples) and GSE36001 (comprising 19 osteosarcoma samples and 6 normal samples) datasets are merged to be the training group while the GSE126209 (comprising 12 osteosarcoma samples and 11 normal samples) dataset was confirmed as the test group. The Principal Component Analysis (PCA) plots before and after merging the two datasets were generated using “stats” package of R software to examine the comprehensive data representation.

### 2.2 Differentially expressed gene screening

To identify differentially expressed genes (DEGs) between osteosarcoma and normal samples, the two datasets (GSE19276 and GSE36001) were merged as the training set, and DEGs were filtered with |logFC|≥1.0 and FDR<0.05 by utilizing the “limma” package in R software. After extracting the DEGs and their expression in the training set, the “pheatmap” and “ggplot2” packages were utilized to visually draw the “heat map” and “volcano plot” of these DEGs.

### 2.3 Functional enrichment analyses of DEGs

The Gene Ontology (GO; http://www.geneontology.org) comprehensively and computably provided the functions of genes and gene products (The Gene Ontology Consortium, 2019). Kyoto Encyclopedia of Genes and Genomes (KEGG; https://www.kegg.jp) were utilized to integrate various biological pathways on gene and gene products (Kanehisa et al., 2022). Disease Ontology (DO; http://disease-ontology.org) was employed to integrate human diseases and the human genome corresponding to genes (Schriml et al., 2012). We utilized GO, KEGG and DO enrichment analysis by the “clusterProfiler” package to reveal the biological functions, downstream signaling pathways, and human diseases of these DEGs.

### 2.4 Gene set enrichment analysis (GSEA)

Focused on interpreting the shared functions, properties, and regulation of the biological items represented within the datasets, GSEA was performed to identify the biological functions-related items that differed most significantly between the osteosarcoma and the control subgroups. GESA enrichment has become the most curial analytical methods analysis (Subramanian et al., 2005). The GSEA was performed by employing the “clusterProfiler” package of R software.

### 2.5 Machine learning analysis

To further select diagnostic candidates among these DEGs, we adopted two commonplace algorithms LASSO and SVM-RFE. The LASSO algorithm could stabilize the vanilla linear regression and circumvent overfitting and outliers, thereby predicating the accuracy for selecting variables (McEligot et al., 2020). The SVM-RFE algorithm was committed to accurate feature selection, and hence, it is commonplacely utilized to filter out the features and potential disease biomarkers for microarray data (Sanz et al., 2018). In our research, the two algorithms were combined to identify the important DEGs via the “glmnet” package for LASSO and “e1071” package for SVM-RFE. On the basis of LASSO and SVM-RFE algorithms, we use a Venn diagram to visualize the overlapping genes and the final diagnostic candidates.

### 2.6 Diagnostic value of feature biomarkers in osteosarcoma

The receiver operating characteristic (ROC) curve with horizontal coordinate sensitivity and vertical coordinate 1-specificity range from 0–1.0 was employed to evaluate the diagnostic value of each diagnostic biomarker. The calculation area below the ROC curve (AUC) value along with 95% CI was calculated by “pROC” package of R software (Mandrekar, 2010). Additionally, the candidates are considered to have great diagnostic value and considerable accuracy in further research with AUC value > 0.9 in the training group. Next, “rms” and “rmda” packages were used to generate the nomogram comprising all candidates. By using the nomogram’s predictions, the comprehensive ROC curve was constructed to assess the overall model accuracy.

### 2.7 Validation of the diagnostic value and differential expression of feature biomarkers

In order to further validate the diagnostic value and differential expression of feature biomarkers, the GSE126209 dataset as test group was employed for verify the diagnostic value. Simultaneously, we compared the expression of candidate genes between osteosarcoma and normal samples in the test dataset, and plotted a diagram to visualize the outcome.

### 2.8 Prognostic value analysis

In this section, we conducted univariate Cox analysis and Kaplan-Meier survival analysis to explore the prognostic value of screened genes in the prognosis of osteosarcoma patients. Osteosarcoma patients with survival information were extracted from TARGET-OS database samples were obtained from the TARGET database (https://ocg.cancer.gov/programs/target). R packages “survival” and “survminer” were used to conduct this investigation process.

### 2.9 Immune cell infiltration analysis

Increasing evidence has demonstrated that the degree of immune cell infiltration was significantly correlated with cancer progression and the prognosis of cancer patients (Mao et al., 2021). By employing CIBERSORT algorithm, the infiltration of 22 immune cell types in osteosarcoma was illustrated. The “ggplot2” and “pheatmap” packages were utilized to visualize the relationship among these different immune cells. The correlation heatmap was implemented by “corrplot” package while the violin plot was corresponded by “vioplot” package, which showed the difference in immune cell infiltration between the osteosarcoma subgroup and the normal subgroup. Simultaneously, the “ggplot2” and “ggpubr” packages are utilized to analyze the correlation between the functional biomarkers and immune cell infiltration via Spearman correlation analysis.

### 2.10 qRT-PCR

The total RNA of hfob, HOS, U2-OS and 143B was extracted using SteadyPure Rapid RNA Extraction Kit (Accurate Biology, China). The step of reverse transcription of the total RNA into complementary DNA (cDNA) synthesis was conducted by Evo M-MLV Reverse Transcription Kit (Accurate Biology, China). SYBR Green qPCR Hub Mix (Accurate Biology, China) was utilized for quantitative real-time polymerase chain reaction (qRT-PCR). The GAPDH gene is used as an internal reference gene. Conditions for PCR were: one cycle at 95°C for 30 s, 40 cycles of denaturation for 5 s at 95°C followed by amplification for 30 s at 60°C. The primer sequences of ASNS, CD70, SRGN and TRIB3 can be found in of the Supplementary Material.

### 2.11 Western blotting

Western blot was used to detect the expression levels of ASNS, CD70 and TRIB3 proteins in hfob, HOS and U2OS cell lines. The protein was extracted using RIPA lysate and the concentration of the protein was measured using BCA method. We added protein solution in a 4:1 ratio to 5 × Sample the buffer solution and denatured it in 100°C for 15 min. We performed 10% SDS-PAGE electrophoresis, and transferred the PVDF membrane for an hour. Then the membrane was placed in a TBST incubator, and with skimmed milk at room temperature, and sealed for 2 h. We added diluted first antibodies, ASNS (R22614, Chengdu Zen Biotechnology), TRIB3 (R383249, Chengdu Zen Biotechnology) and CD70 (AF5265, Affinity), and then incubated them overnight on a shaking bed at 4°C. We used TBST to elute 3 times for 5 min each time. The secondary antibody was diluted with TBST in a ratio of 1:5,000, incubated at room temperature for 2 h and then colored using ECL method. Finally, we used Image Lab software to analyze the grayscale values of the bands, and the relative expression level of the target protein = the grayscale value of the target protein/Tubulin grayscale value.

### 2.12 Statistical analysis

All analyses above were conducted on R (4.2.1) and Perl software. All experiments were carried out in replicates of three times. Differential comparisons between two subgroups were analyzed by Student’s t-test. *p*-value < 0.05 was considered statistically significant.

## 3 Results

### 3.1 Identification of DEGs in osteosarcoma

**FIGURE 1:**
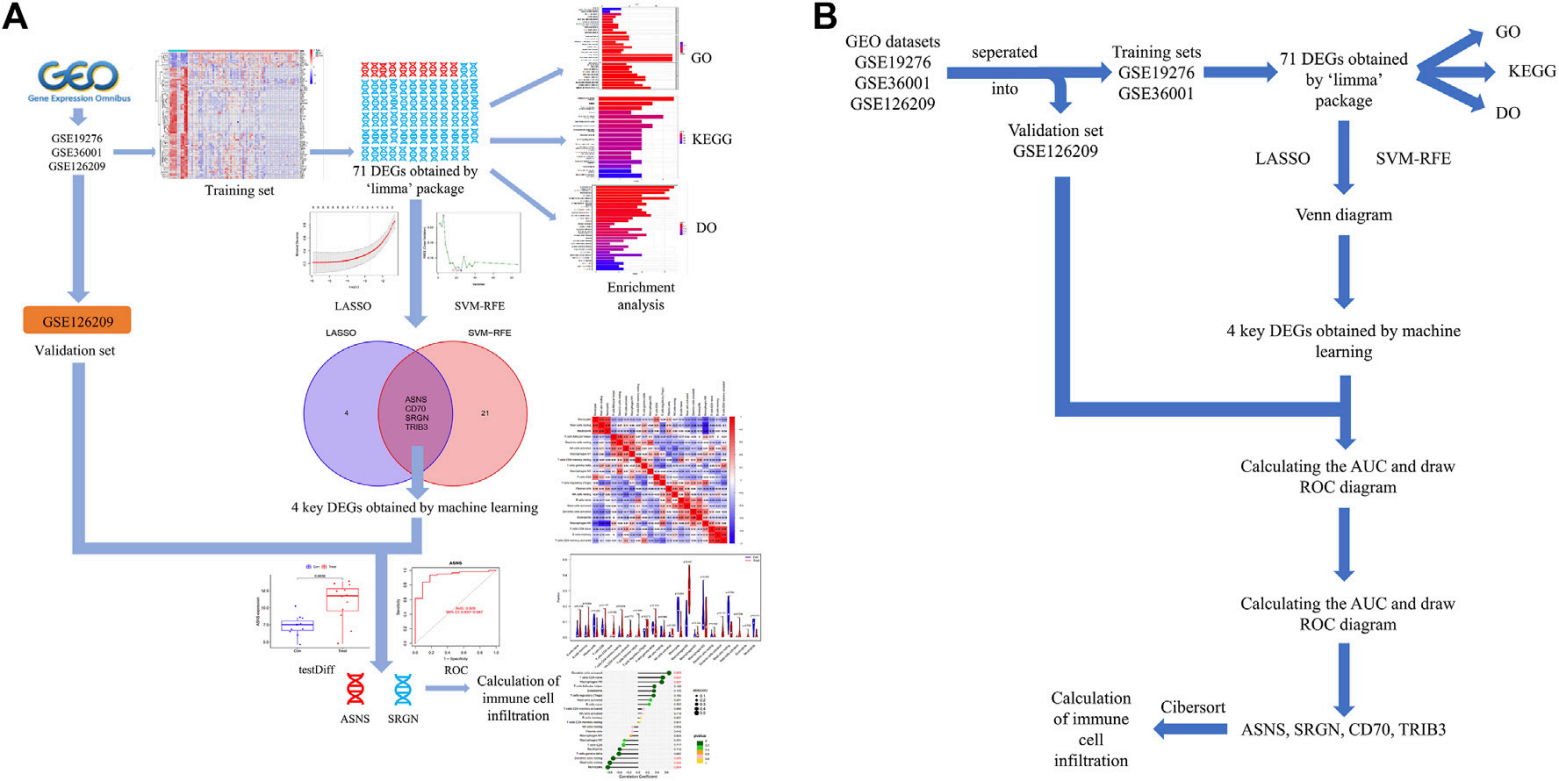
**(A)** Flowchart of this present study. **(B)** Pipeline flow chart of the study.

**FIGURE 2:**
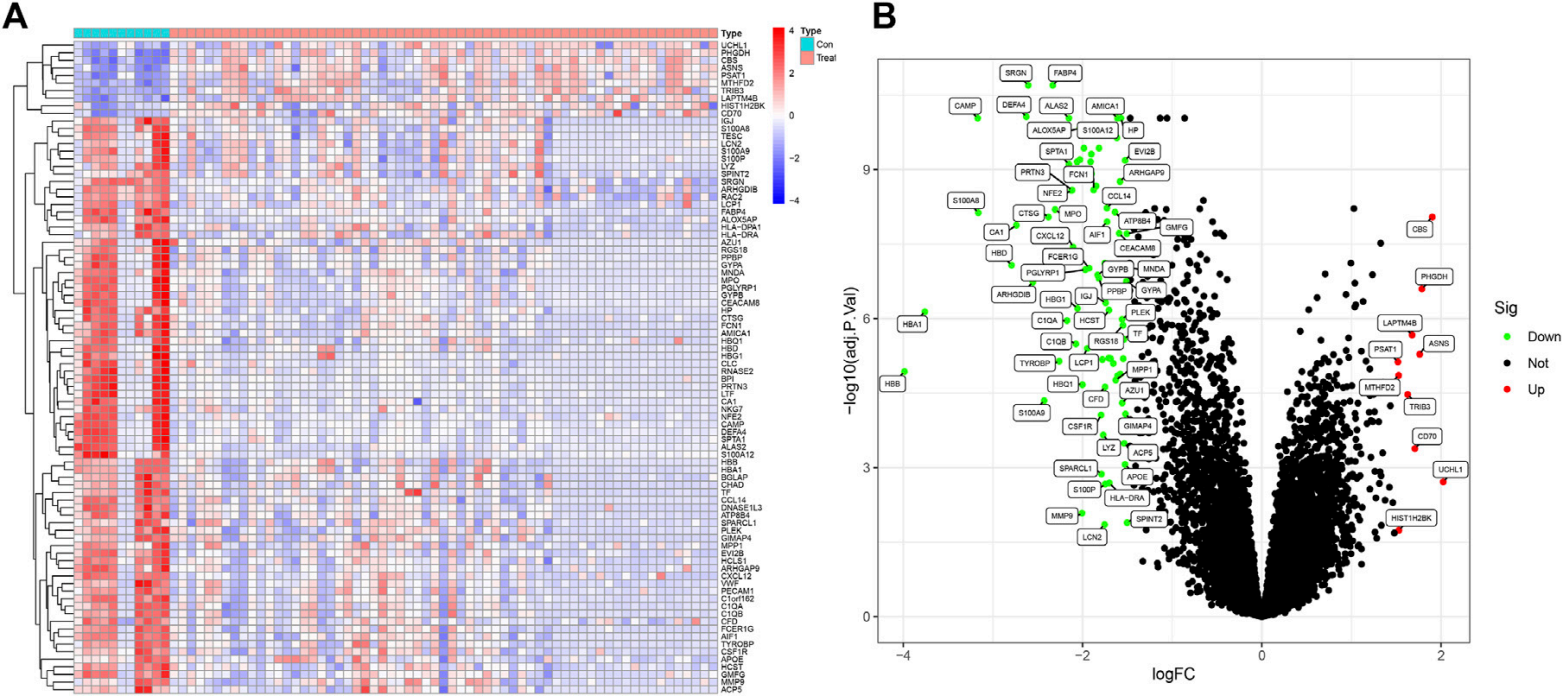
DEGs between osteosarcoma samples and normal samples in the training dataset **(A)** The heatmap of DEGs. **(B)** The volcano plots of DEGs, (GSE19276, 36001).

The workflow of this investigation was shown in Figure 1. A total of 71 DEGs were confirmed in the GEO osteosarcoma merged dataset by employing the limma method, of which 10 genes were upregulated and 61 genes were downregulated. The heatmap and volcano plot of these detailed DEGs between osteosarcoma samples and normal samples are shown in Figures 2A, B. The principal component analysis (PCA) plot (Supplementary Figure S1) indicated that batch effects between samples had been removed after correction.

### 3.2 Functional enrichment analyses of DEGs

**FIGURE 3:**
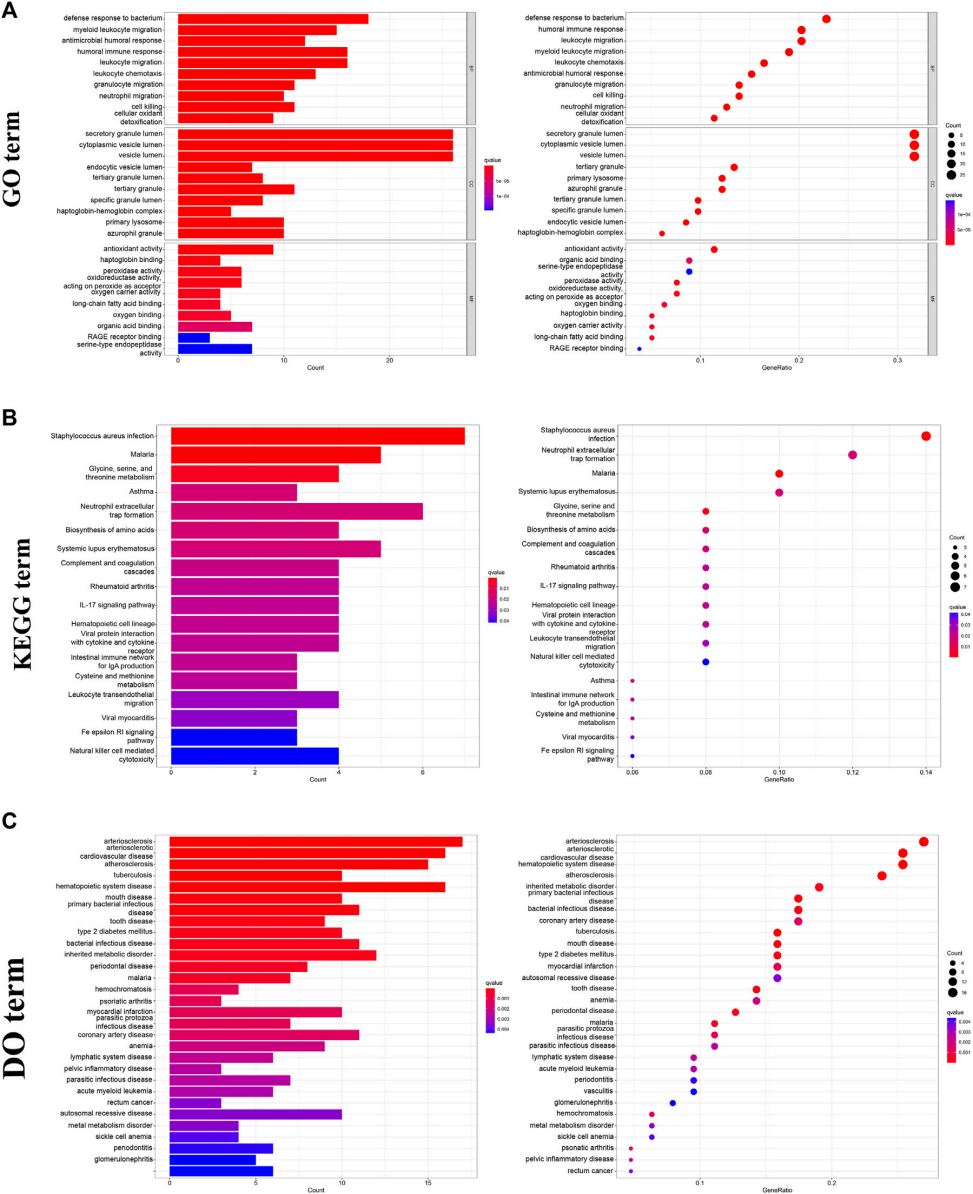
Functional enrichment analysis of DEGs. **(A)** GO analysis of DEGs, including BP, CC, and MF. **(B)** KEGG analysis of DEGs. **(C)** DO analysis of DEGs, (GSE19276, 36001).

In furtherance of identifying the potential function and biological processes among these DESs, GO, KEGG and DO were utilized to analysis. The results of GO analysis revealed that the major enrichment of these DEGs on biological process (BP) were defense response to bacterium, humoral immune response, and leukocyte migration, suggesting the influence of DEGs on the immune system. As for the cellular component (CC) ontology, DEGs were mainly enriched in intracellular membranous structures, counting secretory granule lumen, and cytoplasmic vesicle lumens. Molecular function (MF) analysis showed that antioxidant activity, serine-type endopeptidase, and organic acid were identified (Figure 3A). Simultaneously, the KEGG analysis showed that bacterial infections, immune processes, and malaria were the most enriched items (Figure 3B). With regard to DO research, it was demonstrated that these DEGs were most correlated with cardiovascular disease, including arteriosclerosis, arteriosclerotic, cardiovascular disease and hematopoietic system disease, etc. (Figure 3C).

### 3.3 GSEA enrichment analysis

**FIGURE 4:**
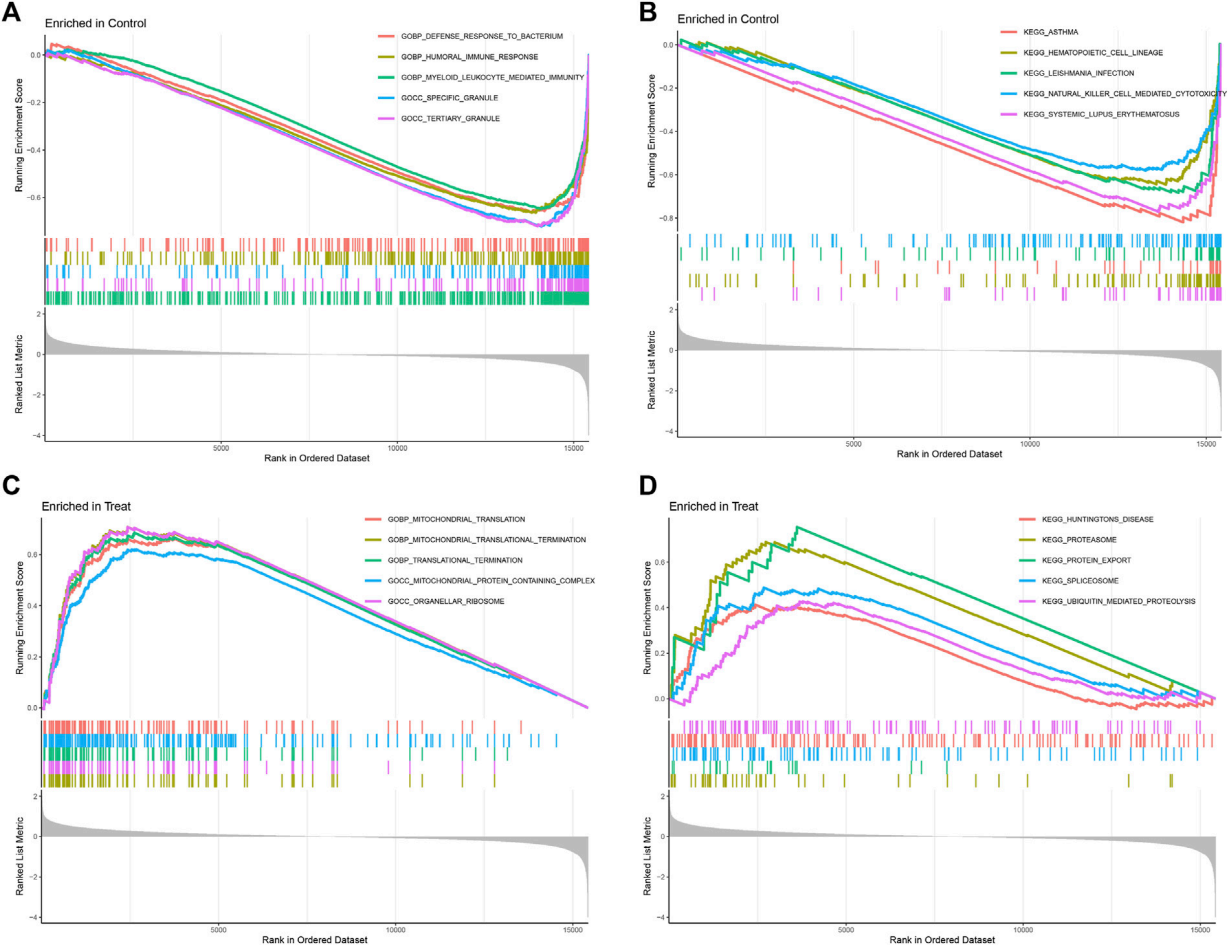
Gene set enrichment analysis (GSEA) of GOBP **(A,B)** and KEGG **(C,D)** between control and treatment groups, (GSE19276, 36001).

In GESA enrichment analysis, including KEGG and Gene Ontology Biological Process (GOBP), our results of GOBP showed that DEGs were most enriched in defense response to bacterium, humoral immune response, myeloid leukocyte mediated immunity, specific granule and tertiary granule in normal samples. In osteosarcoma samples, mitochondrial translation, mitochondrial translational termination, translational termination, mitochondrial protein containing complex and organellar ribosome were mostly enriched GOBP items, indicating the potential biological functions of these genes in mediating mitochondrial functions during osteosarcoma initiation and progression (Figures 4A, C). With regards to KEGG analysis, asthma, hematopoietic cell lineage, leishmania infection, NK cell mediated cytotoxicity and systemic lupus erythematosus were enriched downstream pathways of DEGs in normal samples, while huntingtins disease, proteasome, protein export, spliceosome and ubiquitin mediated proteolysis were potential signaling pathways modulated by DEGs in osteosarcoma samples (Figures 4B, D).

### 3.4 Identification of candidate diagnostic genes via machine learning

**FIGURE 5:**
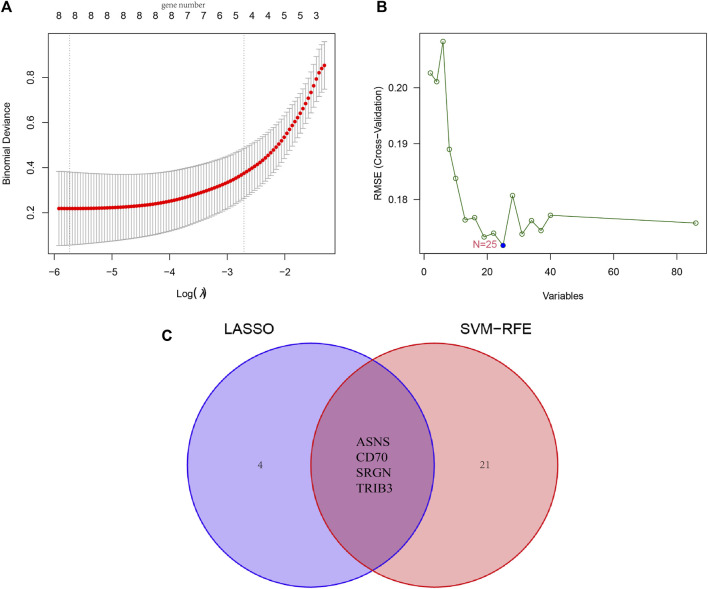
Identification of novel diagnostic biomarkers for patients with osteosarcoma by machine learning. **(A)** Tuning feature selection in the LASSO. **(B)** A plot of diagnostic markers selected by the SVM-RFE algorithm. **(C)** The Venn diagram of four diagnostic markers shared by the LASSO and SVM-RFE algorithms, (GSE19276, 36001).

Furthermore, we combined LASSO liner regression and SVM-RFE algorithms to select critical candidate genes with promising diagnostic values from the above-obtained DEGs. There was a total of eight candidate biomarkers being captured by the LASSO algorithm, and 25 outputs were identified based on the SVM-RFE algorithm (Figures 5A, B). Then, a total of four genes (*ASNS*, *SRGN*, *CD70*, *TRIB3*) were yielded by intersecting the two baskets of genes from LASSO and SVM-RFE algorithm, and the results were visualized by Venn image (Figure 5C).

### 3.5 Diagnostic value analysis

**FIGURE 6:**
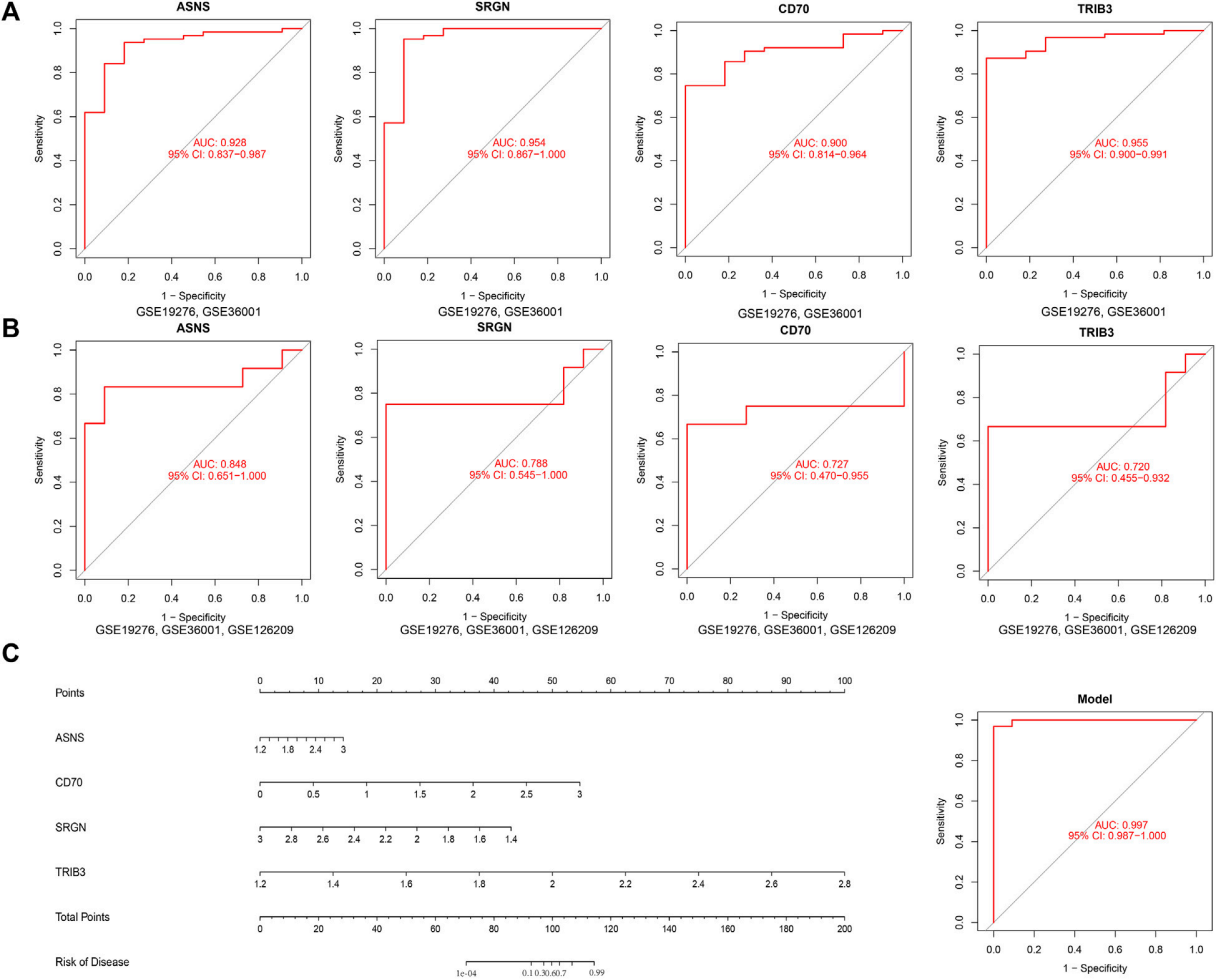
Investigation of the diagnostic efficacy of four biomarkers for osteosarcoma. **(A)** ROC curves of ASNS, SRGN, CD70, and TRIB3 in the training set, (GSE19276, 36001) **(B)** ROC curves of ASNS, SRGN, CD70, and TRIB3 in the validation set, (GSE19276, 36001, 126209). **(C)** Comprehensive ROC curve of the model.

To further examine the potential of these four candidate genes in diagnosing osteosarcoma, we employed a ROC curve to evaluate the diagnostic specificity and sensitivity of each gene with the AUC and 95% CI. Conforming to the ROC curves in Figure 6A, the results were as follows: *ASNS* (AUC 0.928, 95% CI 0.837–0.987), *SRGN* (AUC 0.954, 95% CI 0.867–1.000), *CD70* (AUC 0.900, 95% CI 0.814–0.964), *TRIB3* (AUC 0.955, 95% CI 0.900–0.991), indicating that all 4 genes had high AUC value than 0.90 and possessed excellent diagnostic value for osteosarcoma patients (Figure 6A). Furthermore, we constructed a nomogram comprising all the four candidates. By using the nomogram’s predictions, the comprehensive ROC curve was constructed to assess the overall model accuracy. In Figure 6C, the comprehensive ROC curve showed the results: AUC 0.997, 95% CI 0.987–1.000. The results demonstrated the excellent performance of the model in diagnosing osteosarcoma patients.

### 3.6 Validation of the diagnostic value

**FIGURE 7:**
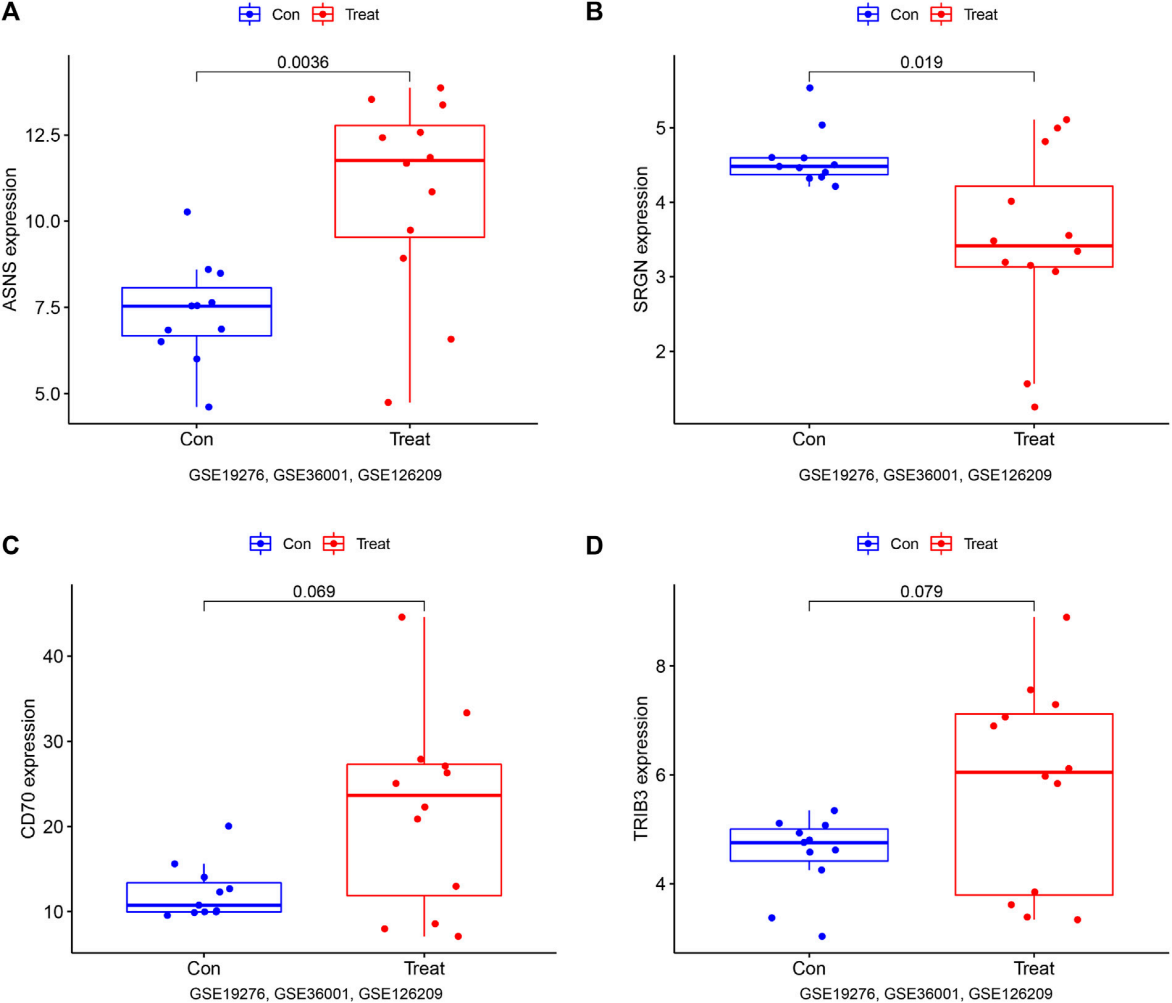
Box plots of differential expression of ASNS **(A)** SRGN **(B)**, CD70 **(C)**, and TRIB3 **(D)** in the validation set, (GSE19276, 36001, 126209).

To further verify the diagnostic value of these genes in osteosarcoma, we also established the ROC curve in the testing dataset in Figure 6B. The AUC and 96% CI of each gene were as follows: *ASNS* (AUC 0.848, CI 0.651–1.000), *SRGN* (AUC 0.788, CI 0.545–1.000), *CD70* (AUC 0.727, CI 0.470–0.955), and *TRIB3* (AUC 0.722, CI 0.455–0.932). These findings further confirmed these candidate genes all possessed high diagnostic value for osteosarcoma patients. In furtherance of obtaining more credible results, we also compared the differential expression of *ASNS*, *SRGN*, *CD70,* and *TRIB3* between the normal samples and osteosarcoma samples in the testing dataset in Figures 7A–D. The results showed that the expression of *ASNS* (*p* = 0.0036), *CD70* (*p* = 0.019) and *TRIB3* (*p* = 0.069) expression was upregulated while *SRGN* (*p* = 0.079) expression was slightly downregulated in osteosarcoma samples compared with normal samples, which was consistent with the findings in the training dataset.

### 3.7 Prognostic value analysis

To further investigate the relationship between the expression of these candidates and survival outcomes in osteosarcoma, we employed the Cox proportional hazards model and Kaplan-Meier curves. Our results (Supplementary Figure S2) found that osteosarcoma patients with high TRIB3 (*p* = 0.017) and SRGN (*p* = 0.010) expression levels had longer overall survival times than those with low expression. In addition, the overall survival time of osteosarcoma patients with high ASNS expression was shorter than those with low expression of ASNS, but without reaching statistical significance (*p* = 0.076). While the OS of osteosarcoma patients with high expression of CD70 was higher than that of patients with low expression of CD70 without reaching statistical significance (*p* = 0.108). The univariate Cox analysis results (Supplementary Figure S3) indicated that SRGN was a protective factor for osteosarcoma patients with hazard ratio (95% CIs) of 0.733 (0.546–0.985) (*p* = 0.039). The HRs (95% CIs) of ASNS, CD70 and TRIB3 were 1.128 (0.784–1.623) (*p* = 0.516), 0.893 (0.583–1.370) (*p* = 0.605), 0.840 (0.542–1.301) (*p* = 0.435). These findings confirmed that several candidates, such as SRGN and TRIB3 may function as promising prognostic biomarkers for osteosarcoma patients.

### 3.8 Immune infiltration analysis

**FIGURE 8:**
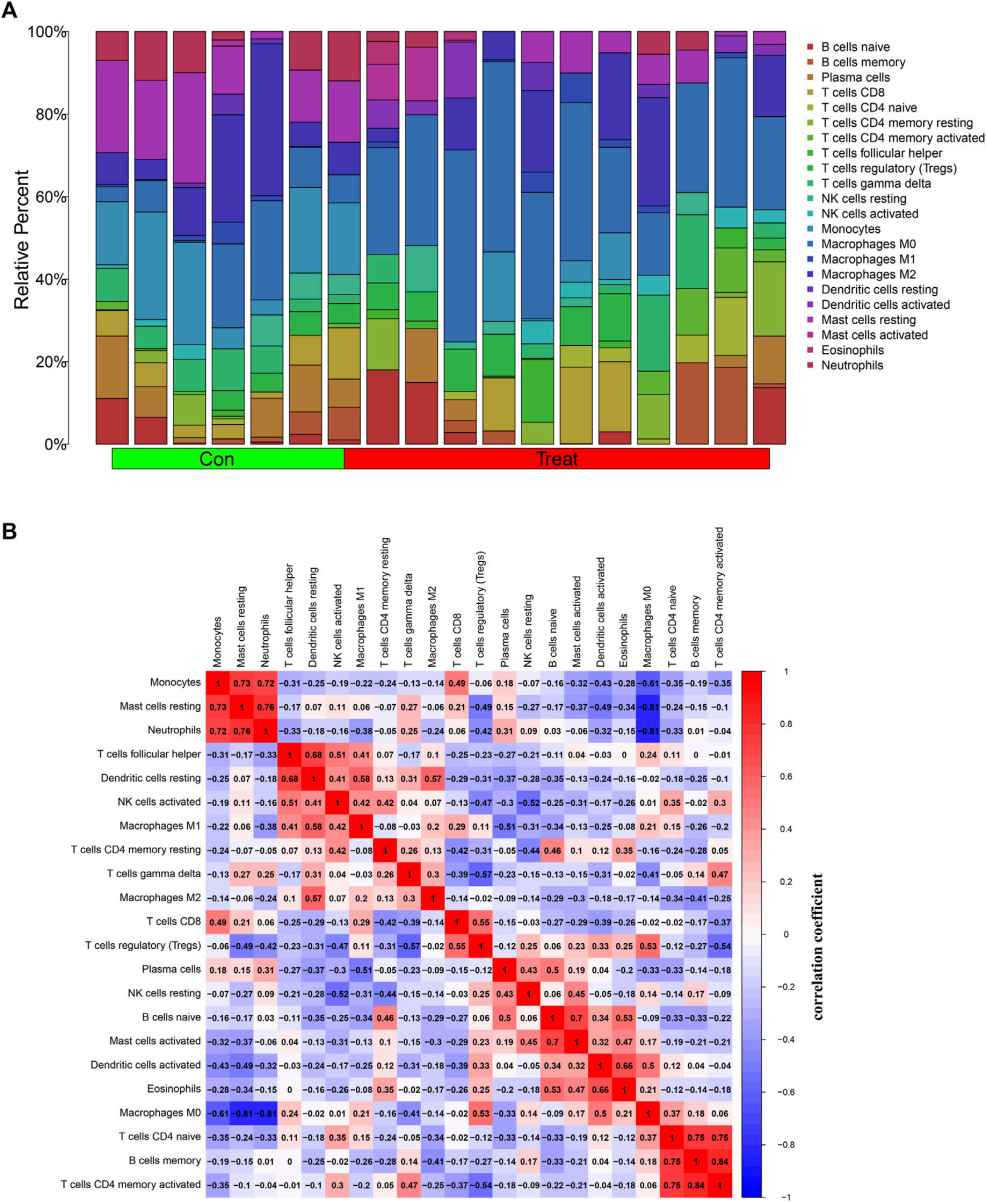
Investigation of immune cell infiltration in osteosarcoma by the CIBERSORT algorithm. **(A)** Bar plot of the relative proportions of immune cells. **(B)** The heatmap of correlations between 22 immune cell types; red indicated a positive correlation and blue indicated a negative correlation between two immune cells. **(C)** Violin plot of the differential infiltration of immune cells between osteosarcoma and normal samples.

Previous results showed that DEGs were enriched in several immune-related biological functions and signaling pathways, and the immune microenvironment has been confirmed to play important roles in osteosarcoma tumorigenesis and progression. Hence, we further investigated the immune cell infiltration in osteosarcoma. The CIBERSORT algorithm was employed to identify the proportions of immune cells in osteosarcoma and normal samples (Figure 8A). Then, we further investigated the interaction among the 22 immune cells in the osteosarcoma immune microenvironment. As the results demonstrated, several immune cells were detected to have a positive correlation with the high coefficient value above 0.7: T cells CD4 memory activated with B cells memory (R = 0.84) and T cells CD4 naïve (R = 0.75), neutrophils with mast cells resting (R = 0.76) and Monocytes (R = 0.72), T cells CD4 naive with B cells memory (R = 0.75), and Mast cells resting with Monocytes (R = 0.73). Simultaneously, negative correlations were also detected between several immune cell types with high coefficient values: macrophages M0 with mast cells resting (R = −0.81) and neutrophils (R = −0.81) and monocytes (R = −0.61), T cells regulatory (Tregs) with T cells gamma delta (R = −0.57) and T cells CD4 memory activated (R = −0.54), and NK cells activated with NK cells resting (R = −0.52) (Figure 8B). In addition, with the results yielded by the CIBERSORT algorithm, the proportions of monocytes (*p* = 0.004), macrophages M0 (*p* < 0.001), mast cells resting (*p* = 0.004), and neutrophils (*p* = 0.012) in osteosarcoma samples were extremely higher than normal samples with all *p* values < 0.05 (Figure 8C). Overall, a variety of immune cells were differentially infiltrated in patients with osteosarcoma, which may serve as the potential therapeutic target in osteosarcoma treatment.

### 3.9 Correlation between DEGs and immune-infiltrating cells

**FIGURE 9:**
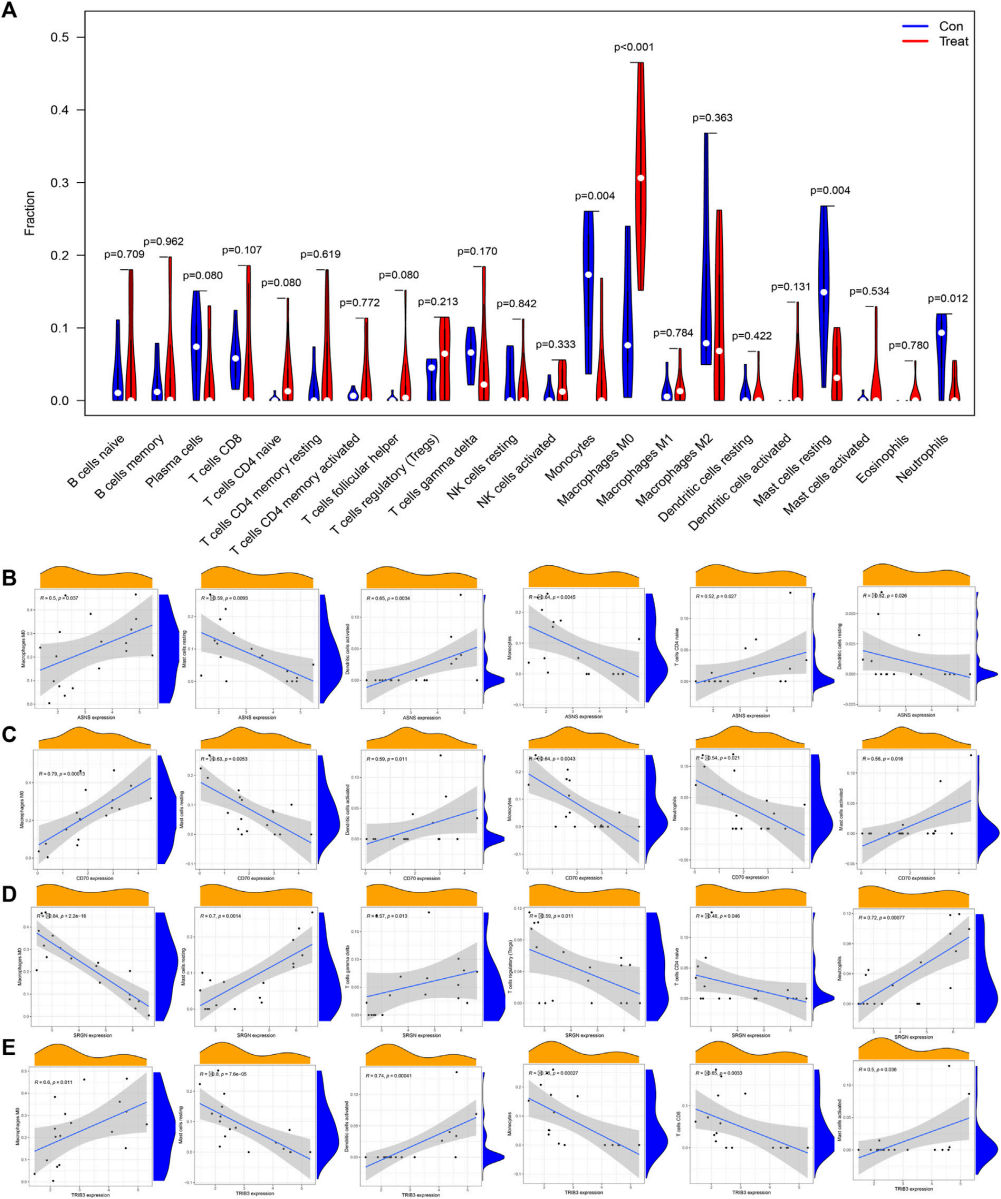
Correlation analysis between diagnostic biomarkers and the infiltration of immune cells in osteosarcoma. **(A)** Lollipop plot of the association of ASNS, SRGN, CD70, and TRIB3 with immune cells. **(B–E)** Correlation plots of the association of ASNS **(B)**, SRGN **(C)**, CD70 **(D)**, and TRIB3 **(E)** with immune cells.

Next, we further illustrated the potential role of these 4 diagnostic biomarkers in mediating the infiltration of immune cells in osteosarcoma. *ASNS* expression was positively correlated with dendritic cells activated (R = 0.65, *p* = 0.0034), T cells CD4 naive (R = 0.52, *p* = 0.027), macrophages M0 (R = 0.5, *p* = 0.037) and was negatively correlated with monocytes (R = −0.64, *p* = 0.0045), Mast cells resting (R = −0.59, *p* = 0.0093), Dendritic cells resting (R = −0.52, *p* = 0.026). While, *CD70* expression was positively with Macrophages M0 (R = 0.79, *p* = 0.00013), Dendritic cells activated (R = 0.59, *p* = 0.011), Mast cells activated (R = 0.56, *p* = 0.016) and was negatively with Monocytes (R = −0.64, *p* = 0.0043), Mast cells resting (R = −0.63, *p* = 0.0053), Neutrophils (R = −0.54, *p* = 0.021). Simultaneously, *SRGN* expression was positively correlated with Neutrophils (R = 0.72, *p* = 0.00077), Mast cells resting (R = 0.7, *p* = 0.0014), T cells delta (R = 0.57, *p* = 0.013), and was negatively correlated with Macrophages M0 (R = −0.84, *p* < 2.2e−16), T cells regulatory (Tregs) (R = −0.59, *p* = 0.011), T cells naïve (R = −0.48, *p* = 0.046). Currently, *TRIB3* was positively correlated with Dendritic cells activated (R = 0.74, *p* = 0.00041), Macrophages M0 (R = 0.6, *p* = 0.011), Eosinophils (R = 0.5, *p* = 0.036) and was negatively correlated with T cells CD8 (R = −0.8, *p* = 7.6e−05), Monocytes (R = −0.76, *p* = 0.00027), T cells CD8 (R = −0.65, *p* = 0.0033) (Figures 9A, B). These results suggested that these diagnostic biomarkers might be significantly correlated with the immune signature, and targeting these biomarkers might be utilized to promote the anti-tumor immune microenvironment in osteosarcoma.

### 3.10 Identification of the expression of diagnostic biomarkers

**FIGURE 10:**
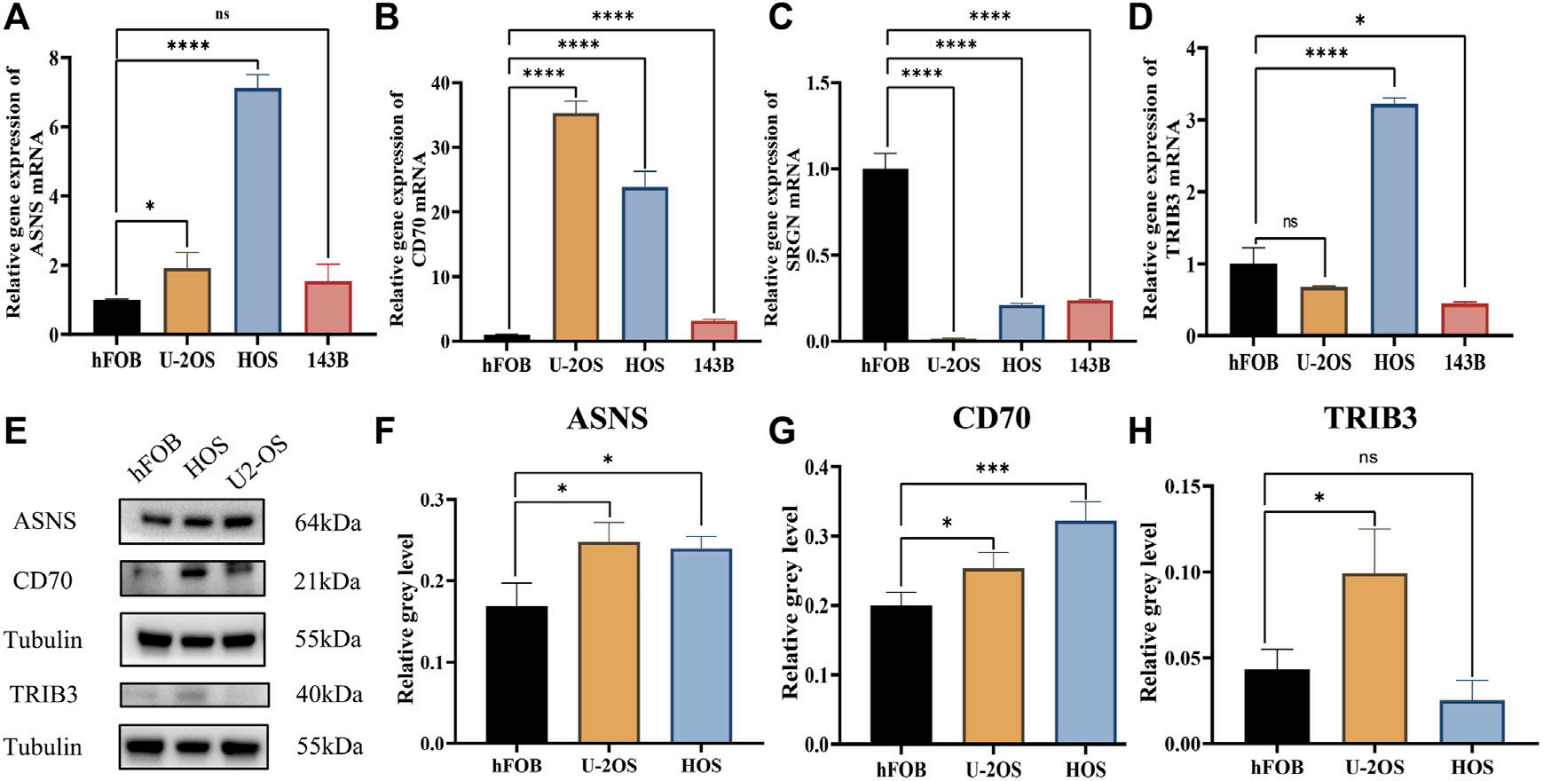
The qRT-PCR for the levels of key genes in control and osteosarcoma cell lines. **(A)** ASNS. **(B)** CD70. **(C)** SRGN. **(D)** TRIB3. The western blot band chart **(E)** and the protein level of **(F)** ASNS, **(G)** CD70 and **(H)** TRIB3.

The qRT-PCR results showed that compared to the control group (hfob), the expression level of CD70 was significantly increased in HOS, U2OS and 143B. The expression level of ASNS was significantly increased in HOS and U2OS, while the trend was not significant in 143B. The significant upregulation of TRIB3 was only detected in HOS cell line. The expression level of SRGN was significantly decreased in all three cell lines compared to the control group (Figures 10A–D).

Furthermore, the western blots results showed that ASNS and CD70 protein levels in both U2OS and HOS cell lines significantly increased compared to the control group, while TRIB3 protein only increased in HOS cell line with statistical significance (Figures 10E–H). These experimental results were basically in line with our bioinformatics analysis results.

## 4 Discussion

Osteosarcoma is the most frequent malignant bone tumor frequently affecting children and adolescents with poor prognosis (Gill and Gorlick, 2021; Meltzer and Helman, 2021). Despite the booming development of therapeutic strategies in osteosarcoma, the 5-year survival of patients with osteosarcoma has hit a plateau over the past decades, and it is urgent to develop effective biomarkers and therapeutic targets (Belayneh et al., 2021; Celik et al., 2022). There have been several studies investigating novel biomarkers for osteosarcoma patients, including lipid metabolism-, ferroptosis-, immune-related genes and so on (Huang W. et al., 2022; Wang et al., 2022; Yang et al., 2022). Nevertheless, machine learning methods have not been applied in screening diagnostic biomarkers for patients with osteosarcoma. In the present study, we combined integrated bioinformatics analysis and machine learning to explore novel diagnostic biomarkers for patients with osteosarcoma as well as their prognostic value and potential functions and roles in mediating osteosarcoma immune microenvironment. Besides, we also validated the expression of diagnostic candidates in osteosarcoma *in vitro*.

Three osteosarcoma microarray datasets were extracted from the GEO online database. A total of DEGs were identified in osteosarcoma. DEGs were mainly enriched in small molecule catabolic process, amino acid metabolic process, and other biological processes. GSEA enrichment results showed that were highly active in. which may be closely related to the initiation and development of osteosarcoma. To improve the diagnostic value and clinical availability of novel diagnostic candidates, the LASSO regression algorithm was employed to minimize regression coefficients to reduce overfitting, and the machine learning SVM-RFE algorithm was employed to achieve the minimal classification error. As a result, a total of five pivotal candidate genes (*ASNS*, *SRGN*, *CD70*, and *TRIB3*) were identified for diagnosing patients with osteosarcoma. According to our results, *ASNS*, *CD70,* and *TRIB3* were upregulated in osteosarcoma patients, while *SRGN* was downregulated in osteosarcoma patients.

Asparagine synthase (*ASNS*) catalyzes the synthesis of asparagine and glutamate from aspartic acid and glutamine in an ATP-dependent amidotransferase reaction (Richards and Kilberg, 2006). *ASNS* has recently received considerable attention in several cancers. *ASNS* is highly methylated in B-cell precursor acute lymphoblastic leukemia (BCP-ALL) patients with favorable karyotypes but is mostly unmethylated in BCP-ALL patients with poor prognostic karyotypes (Watanabe et al., 2020). In NSCLC, it has been found that ATF4 targets *ASNS* to achieve apoptotic suppression, protein biosynthesis, and mTORC1 activation. Moreover, the inhibition of AKT can suppress *ASNS* expression and depletion of extracellular asparagine, subsequently inhibiting tumor growth. Inhibiting *ASNS* through AKT suppression can sensitize cancer cells to L-asparaginase, providing evidence for *ASNS* as a novel therapeutic target in NSCLC (Gwinn et al., 2018). Serglycin (*SRGN*), a major proteoglycan expressed in hematopoietic cells, endothelial cells, and macrophages, has been found to participate in the initiation and progression of human malignancies (Zhang et al., 2017; Ma et al., 2020; Tanaka et al., 2022). For instance, the upregulation of *SRGN* in chemoresistant breast cancer cells, serum and tissue samples from breast cancer patients with poor response to chemotherapy. Mechanistically, *SRGN* can facilitate chemoresistance by cross-talking with the transcriptional coactivator YAP to maintain the stemness of breast cancer cells *in vivo* and *in vitro* (Zhang Z. et al., 2020). However, the expression pattern and biological functions of *SRGN* in multiple cancer types, especially osteosarcoma remain largely unknown.

*CD70* is the natural ligand for the tumor necrosis factor (TNF) superfamily member CD27, which has been identified to induce the B cell and T cell activation. In cancer biology, *CD70* can facilitate immune evasion and tumor progression in the tumor immune microenvironment (Jacobs et al., 2015). The vital functions of the CD70-CD27 pathway in oncology have been investigated over the last decade, and targeting the CD70-CD27 pathway by different approaches has also been illustrated in different malignancies. In osteosarcoma studies, *CD70* has been found to be expressed in a subset of osteosarcoma patients, and *CD70* may represent a novel candidate for antibody-based targeted immunotherapy in these patients with *CD70* (+) osteosarcoma (Pahl et al., 2015). In addition, PLCE1 may induce immune escape in osteosarcoma through the CD70-CD27 signaling pathway (Huang L. et al., 2022). Tribbles homolog 3 (*TRIB3*) is a mammalian gene that is upregulated in response to several types of cell death-inducing cellular stress (Ord and Ord, 2017). The functions of *TRIB3* on cancer progression have attracted multiple studies. It has been found that *TRIB3* is positively related to breast cancer stemness and development. Mechanistically, *TRIB3* can facilitate breast cancer stem cells through regulating AKT to interfere with the FOXO1-AKT interaction and inhibit FOXO1 phosphorylation, ubiquitination, and degradation by E3 ligases SKP2 and NEDD4L (Yu et al., 2019). In NSCLC, *TRIB3* has been found to interact with EGFR and recruits PKCα to induce a Thr654 phosphorylation and WWP1-induced Lys689 ubiquitination in the EGFR juxtamembrane region, subsequently promoting EGFR recycling, stability, downstream activity, and NSCLC stemness (Yu et al., 2020). Overall, these four diagnostic biomarkers have been found to be critically involved in cancer initiation and progression, but their roles in osteosarcoma are still largely inconclusive. Identifying the clinical significance and functional roles of these candidates in osteosarcoma may help understand pathological process of osteosarcoma and provide novel therapeutic targets.

Furthermore, our study investigated the potential biological functions and signaling pathways mediated by these diagnostic biomarkers in osteosarcoma. Our findings indicate that these diagnostic biomarkers are mainly involved in immune response, leukocyte function, serine/glycine metabolism and IL-17 signaling pathways in osteosarcoma progression. Several studies have investigated the roles of IL-17 signaling pathways in osteosarcoma. IL-17 can facilitate the susceptibility of osteosarcoma cells to NK cell lysis, and IL-17A/IL-17RA interaction can promote osteosarcoma cells metastasis, indicating that targeting IL-17 may be a novel promising strategy to treat osteosarcoma patients (Honorati et al., 2003; Wang et al., 2013). The CD27 and CD70 costimulatory pathway has been found to inhibit the transcription of the key effector molecules IL-17 and the chemokine receptor CCR6, subsequently attenuates associated autoimmunity, Cancer cells can reprogramme their metabolism to support cell growth and proliferation. Serine and glycine are biosynthetically linked, and together provide the essential precursors for the synthesis of proteins, nucleic acids, and lipids that are crucial to cancer cell growth. It has been found that serine and glycine are critical metabolites for cancer cells, and metabolic enzymes of serine and glycine biosynthesis are significantly upregulated during cancer progression (Amelio et al., 2014). In osteosarcoma, the mTORC1/serine/glycine metabolic axis has been reported to promote cellular proliferation and the antioxidant ability to environmental stress, thereby resulting in osteosarcoma progression (Wang et al., 2017). To date, few studies have focused on these biological functions of these diagnostic genes, the involvement of these candidates in these biological functions during osteosarcoma initiation and progression require in-depth investigation in future studies.

Multiple lines of evidence have suggested the critical roles of diverse immune cells in the tumor immune microenvironment in osteosarcoma. In the present investigation, we found that osteosarcoma patients had a higher level of M0 macrophages, whereas a lower level of monocytes, mast cell resting, and neutrophils. Previous studies have indicated that the abundance of monocytes was lower in osteosarcoma, and was significantly correlated with the poor prognosis of osteosarcoma patients (Chen et al., 2020; Chen and Zhao, 2020). M0 macrophages have been found to be highly infiltrated in osteosarcoma samples, which is also consistent with our findings (Zhang C. et al., 2020). We further investigated the relationship between these hub genes and the abundance of these infiltrating immune cells. Our findings showed that *ASNS*, *CD70*, and *TRIB3* were positively correlated with the abundance of macrophages M0 and dendritic activated cells, while was negatively correlated with the abundance of mast cells resting and monocytes. There was also a positive correlation between *ASNS* and CD4^+^ T cells, and a negative correlation between *ASNS* and dendritic resting cells. Our results found that *SRGN* was positively associated with the infiltration of T cells gamma delta, T regulatory cells, and CD4^+^ T cells. In addition, *TRIB3* was a negative modulator of CD8^+^ T cells in osteosarcoma. Previous research has confirmed that *TRIB3* can inhibit CD8^+^ T cell infiltration and stimulate the immune evasion through inhibiting the STAT1-CXCL10 axis in colorectal cancer, which is consistent with our results (Shang et al., 2022). These findings indicate that these candidate genes may function as modulators of T cell infiltration in osteosarcoma, thereby regulating osteosarcoma progression and clinical outcomes. Therefore, targeting these biomarkers may be an effective method to modulate the immune cell infiltration in osteosarcoma.

There are still several limitations in the present research. First, although we included three osteosarcoma datasets, the samples remained few, especially the limited sample size in the validation cohort. The results should be further identified in a more large-scale cohort in the future, which is time-consuming but meaningful. We have also validated the expression pattern of these candidates *in vitro*. Second, although we illustrated the correlations of these candidate hub genes with the abundance of immune cells in osteosarcoma was presented, the exact functions and mechanisms of these genes in mediating osteosarcoma are still worth investigating.

## 5 Conclusion

Our research systematically discovered and verified four immune-associated candidate hub genes (*ASNS*, *SRGN*, *CD70*, *TRIB3*) with high predictive value for diagnosing osteosarcoma by bioinformatics analysis and machine learning algorithms. Besides, we also illustrated the dysregulated immune cell proportion in osteosarcoma, and the potential regulatory functions of these diagnostic hub genes in the tumor immune microenvironment in osteosarcoma, thus providing novel immune-related diagnostic candidate genes for patients with osteosarcoma.

## Data Availability

The datasets presented in this study can be found in online repositories. The names of the repository/repositories and accession number(s) can be found in the article/Supplementary Material.

## Supplementary material

SUPPLEMENTARY FIGURE S1 PCA plot before **(A)** and after **(B)** the batch effect between GSE19276 and GSE36001 was removed.

SUPPLEMENTARY FIGURE S2 Kaplan–Meier estimated overall survival in osteosarcoma patients stratified by ASNS **(A)**, CD70 **(B)**, SRGN **(C)** and TRIB3 **(D)**.

SUPPLEMENTARY FIGURE S3 Forest plot of studies evaluating the hazard ratios of ASNS, CD70, SRGN and TRIB3 for overall survival of patients with osteosarcoma.
