# Supplementary figures and images for: Identification and validation of novel biomarkers associated with immune infiltration for the diagnosis of osteosarcoma based on machine learning
Source: Front Genet. 2023 Sep 4;14:1136783. doi: 10.3389/fgene.2023.1136783 (PMC10507254; doi:10.3389/fgene.2023.1136783)

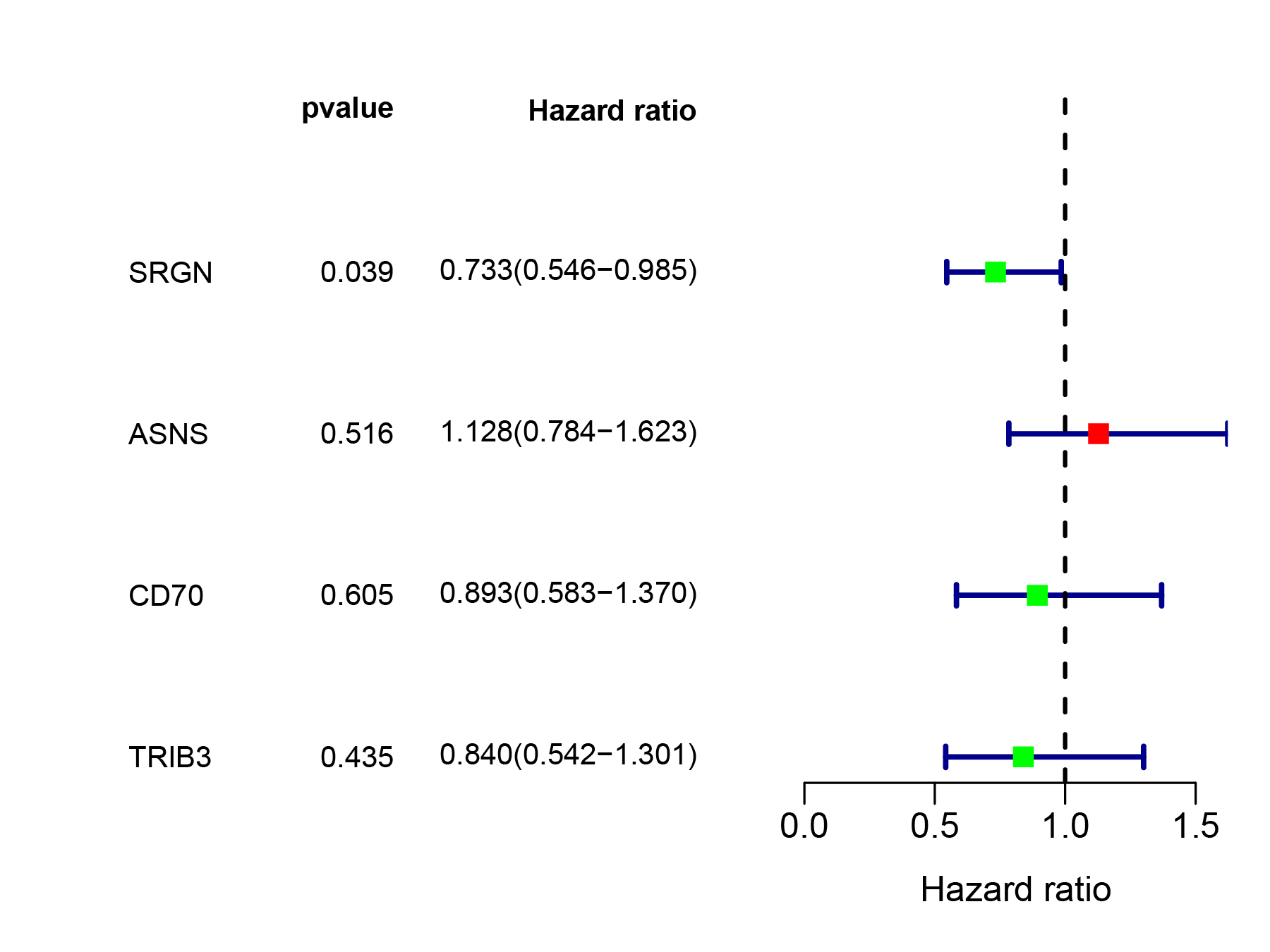

Supplement: Supplementary file 2 [file Image3.JPEG]

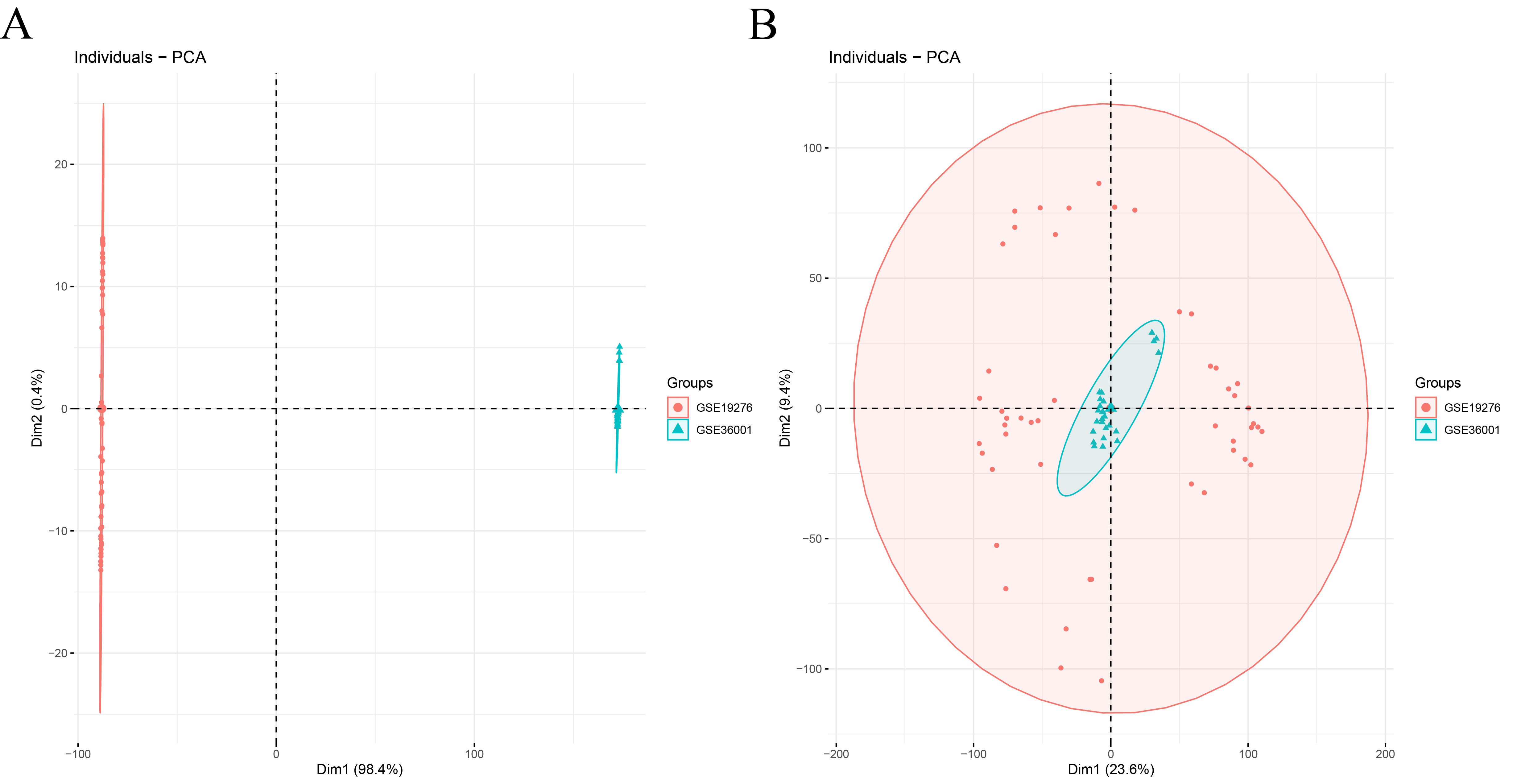

Supplement: Supplementary file 5 [file Image1.JPEG]

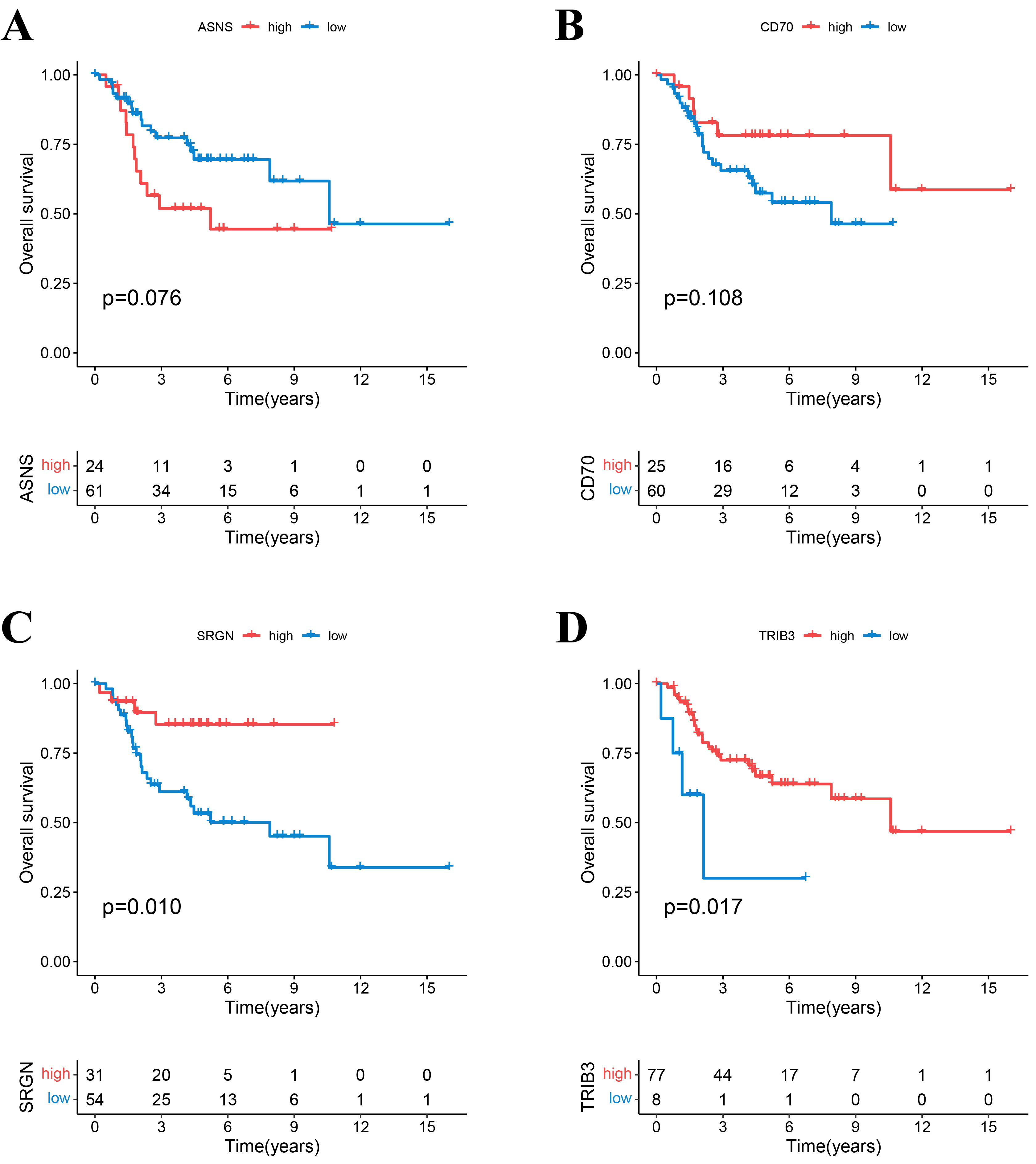

Supplement: Supplementary file 7 [file Image2.JPEG]
